# Incidental Adrenal Masses: A Case Report of an Adrenal Oncocytoma

**DOI:** 10.7759/cureus.47994

**Published:** 2023-10-30

**Authors:** Bruno Giesteira, Jessica Sousa, João Pinheiro Amorim

**Affiliations:** 1 Radiology, Centro Hospitalar Universitário de Santo António, Porto, PRT

**Keywords:** retroperitoneal lesions, radiology-pathology correlation, adrenal masses, incidental adrenal findings, oncocytima

## Abstract

A 59-year-old woman underwent an abdominal and pelvic computed tomography (CT) scan to rule out non-obstructive urolithiasis. The patient was asymptomatic, with the exception of occasional bilateral low back pain. A physical examination did not reveal any notable findings.

The CT scan revealed the presence of an incidental solid left adrenal lesion, which displaced the body of the pancreas and the left kidney. The lesion measured 7 cm × 6.5 cm and exhibited a rounded morphology with well-defined margins. It showed progressive and heterogeneous contrast uptake. Additionally, magnetic resonance imaging (MRI) was performed, confirming the presence of an adrenal lesion with intense and heterogeneous hypersignal on T2. The lesion also demonstrated heterogeneous and persistent enhancement in a dynamic study. Furthermore, there were some indistinct and non-specific hypointense areas identified on both T1 and T2 sequences. The lesion exhibited moderately restricted diffusion.

Although the imaging features were non-specific, there were no indications of invasion or distant metastasis, which made a benign large adrenal mass the most likely diagnosis. Non-functioning pheochromocytoma, lipid-poor adrenal adenoma, as well as metastasis or primary adrenal carcinoma, were considered differential diagnoses.

The patient underwent an elective adrenalectomy, during which the identified lesion was completely resected. The patient's postoperative recovery was uneventful, and she was discharged three days after the procedure. Subsequent histopathological evaluation revealed an oncocytic neoplasm of the adrenal cortex - specifically, an oncocytoma.

## Introduction

Oncocytic neoplasms, or oncocytomas, are usually benign tumors, mostly arising in the kidneys, thyroid, parathyroid, salivatory, or pituitary glands [1]. Oncocytic neoplasms of the adrenal gland are extremely rare, mostly benign and nonfunctional, and in most cases described as an incidental finding [2].

To date, only around 150 cases have been described [3]. There is no precise age distribution, with a tendency to be more frequent in females and in the left adrenal gland [4]. Since most adrenal oncocytic neoplasms are nonfunctional, most patients are asymptomatic, with normal physical examination and without laboratory abnormalities [4].

Adrenal oncocytomas should be characterized with computed tomography (CT) and magnetic resonance imaging (MRI) after the administration of intravenous contrast agents [2]. CT and MRI features of adrenal oncocytic neoplasms are non-specific, making the distinction between oncocytic neoplasms and other benign adrenal lesions a diagnostic challenge. Therefore, the final diagnosis is often based on pathological characterization, typically after surgical resection [4].

## Case presentation

A 59-year-old woman performed an abdominal and pelvic computed tomography without intravenous contrast administration to exclude non-obstructive urolithiasis. The patient was asymptomatic, with no relevant medical or surgical history and no usual medication, with the exception of sporadic use of non-steroidal anti-inflammatory drugs due to occasional bilateral low back pain. The physical examination was unremarkable.

**Figure 1:**
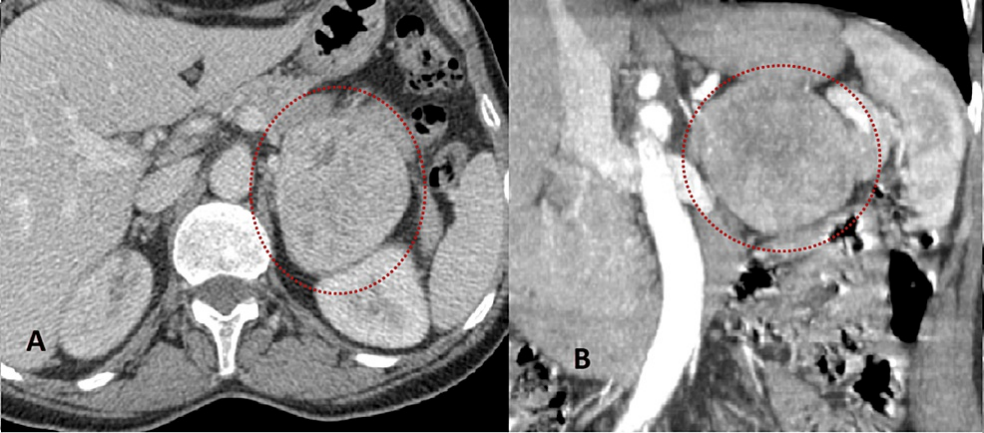
Coronal (A) and axial (B) images of abdominal CT, in arterial (A) and portal venous phases (B). Left adrenal lesion (red circles), measuring 7 cm × 6.5 cm, with rounded morphology and well-defined margins, with heterogenous and progressive enhancement in, with internal ill-defined hypoenhancing areas.

The CT performed incidentally identified a solid retroperitoneal lesion measuring approximately 7 cm with non-specific features in the technical protocol, leading to further characterization by another CT with the administration of iodinated contrast. This CT examination (Figure 1) showed a retroperitoneal neoplasm with a probable starting point in the left adrenal gland, adjacent to and deviating from the body of the pancreas and the left kidney. The lesion measured 7 cm × 6.5 cm with rounded morphology and well-defined margins, with progressive and heterogeneous uptake of contrast (density on the unenhanced phase was 29 HU, on the arterial phase was 94 HU, and on the portal venous phase was 207 HU).

**Figure 2:**
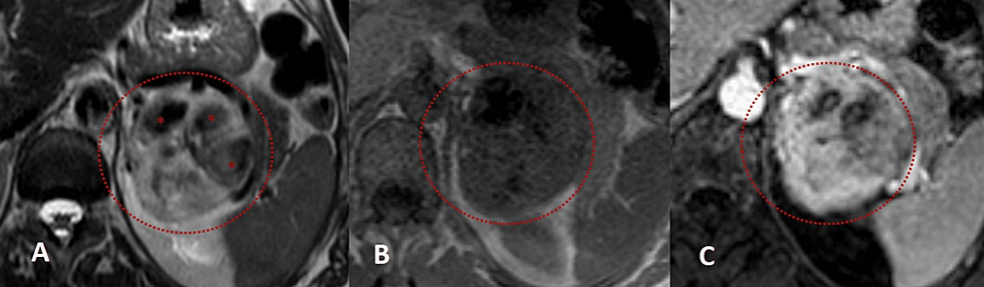
Abdominal magnetic resonance imaging: axial T2-weighted sequence (A), axial in-phase T1-weighted sequence (B), and axial dynamic sequence, portal venous phase (C). Left adrenal solid mass (circles), measuring 7 cm × 6.5 cm, with diffuse high and heterogeneous signal on T2, with internal areas (*) of low signal (A), with diffuse and heterogeneous hyposignal on T1 in-phase sequence (B). After administration of paramagnetic contrast, the lesions showed intense and heterogeneous enhancement (C).

**Figure 3:**
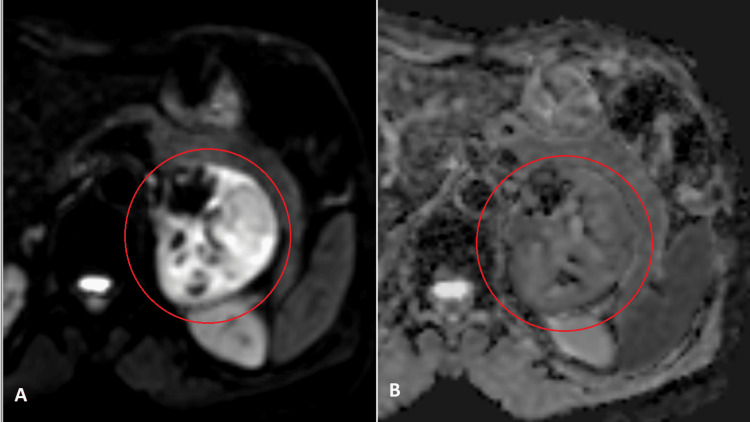
Axial diffusion-weighted imaging study, abdominal MRI. Left adrenal solid mass (circles) with diffuse and intense hypersignal on diffusion-weighted imaging for b values of 1000 (A) and corresponding diffuse moderate hyposignal on ADC map, showing restricted diffusion.

In addition, magnetic resonance imaging was performed, which confirmed the presence of an adrenal lesion with high T2 signal and heterogeneity (Figure 2A), diffuse hyposignal on T1 (Figure 2B), and heterogeneous and persistent enhancement after contrast (Figure 2C). Some ill-defined and nonspecific hypointense areas are identified on both T1 and T2 sequences. There is also moderately restricted diffusion on the lesion (Figure 3).

Despite the nonspecific imaging features, there were no imaging criteria of invasion or distant metastasis, making a non-functioning pheochromocytoma the most likely diagnostic hypothesis, with a differential diagnosis of lipid-poor adenoma, metastasis, or primary adrenal carcinoma.

The patient underwent an elective adrenalectomy, where the lesion was identified and completely resected. The postoperative course was unremarkable, and the patient was discharged three days after the procedure.

**Figure 4:**
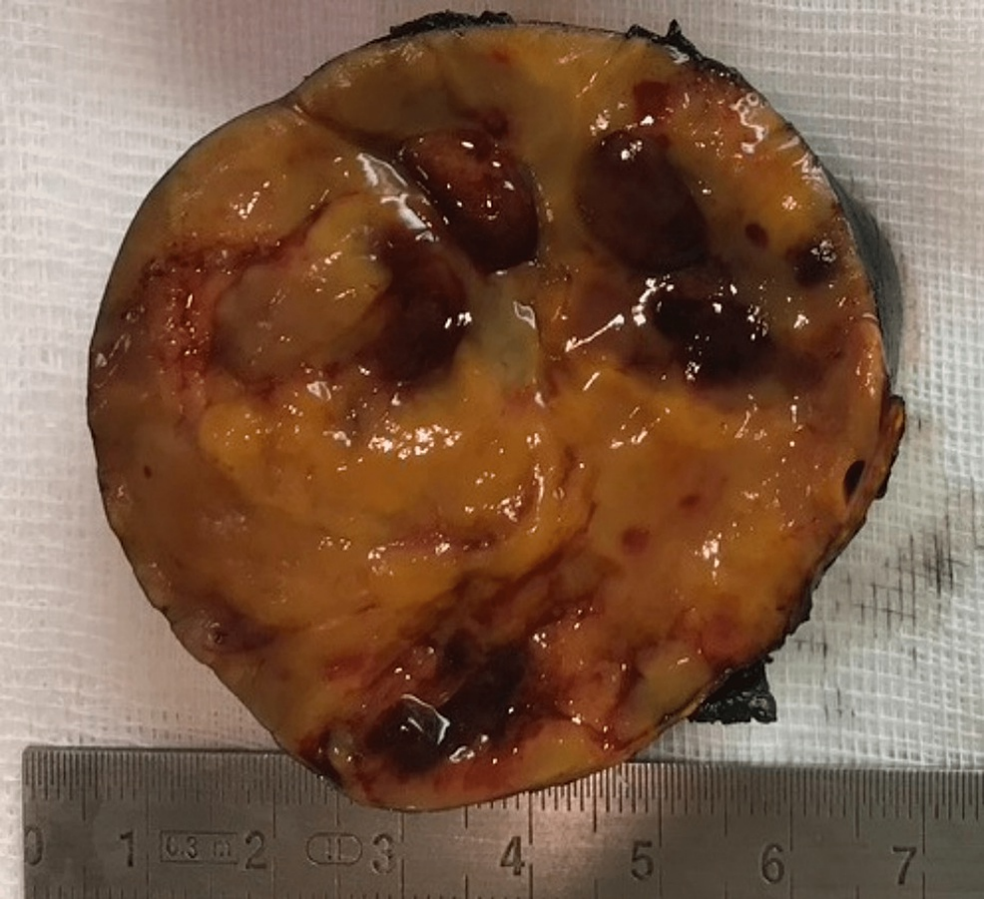
Gross specimen of the resected left adrenal mass. The adrenal solid neoplasm measures 7 cm × 6 cm × 5.5 cm; it is encapsulated with yellow-orange tissues and with foci of hemorrhage.

Further histopathological evaluation revealed, on gross pathology, an expansive adrenal mass measuring 7 cm × 6 cm × 5.5 cm. Upon section, the neoplasm was surrounded by residual glandular parenchyma; it is encapsulated, solid, with yellow-orange tissues, and with foci of hemorrhage (Figure 4).

**Figure 5:**
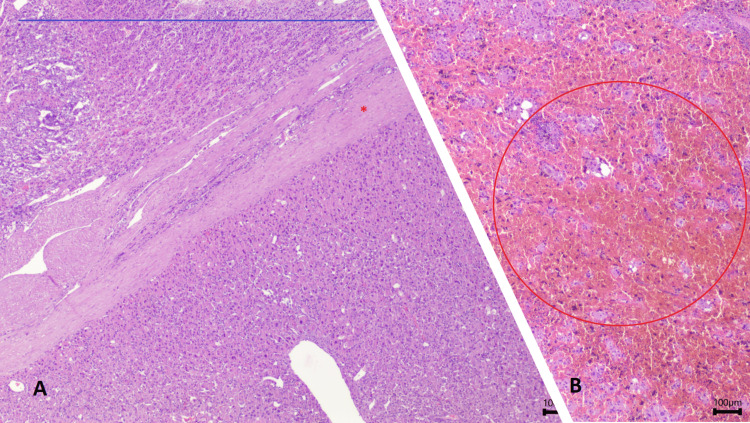
Microscopic pathology study, H&E, 20× magnification. Image (A) shows adrenal gland parenchyma (blue line) is observed to involve an encapsulated (red star) neoplasm of predominantly solid pattern, consisting of cells with an oncocytic phenotype. Image (B) revealed signs of old hemorrhage (red circle), without necrosis or mitotic figures.

Microscopic evaluation revealed a solid pattern of cells with an oncocytic phenotype, ample eosinophilic cytoplasm, a round nucleus, and a nucleolus. The stroma is edematous or focally myxoid. Signs of an old hemorrhage. There is no evidence of necrosis or mitotic figures (Figure 5).

**Figure 6:**
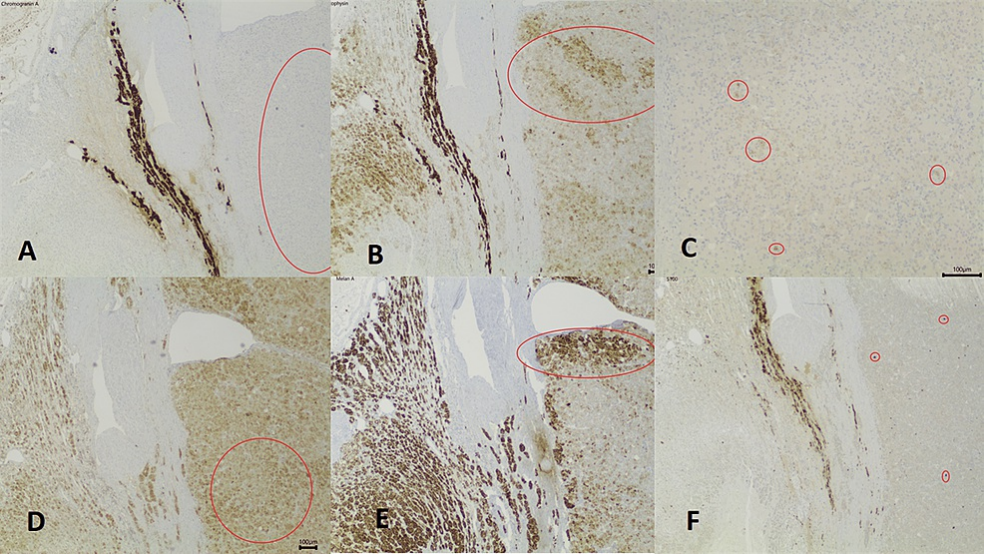
Immunohistochemical study (150× magnification). The neoplastic cells were negative for chromogranin A (red circle) (A), positive for synaptophysin (red circle) (B), focally positive for CAM5.2 (red circles) (C), diffusely positive for calretinin (red circle) (D), positive for MelanA (red circle) (E), and positive for S100, cytoplasmatic and granular (red circles) (F).

In the immunohistochemical study (Figure 6), the neoplastics were focally positive for CAM5.2, diffusely positive for Calretinin, positive for MelanA, Synaptophysin, S100+ (cytoplasmic and granular), and negative for Chromogranin, Inhibin, HMB45, PAX8, and GATA3.

**Figure 7:**
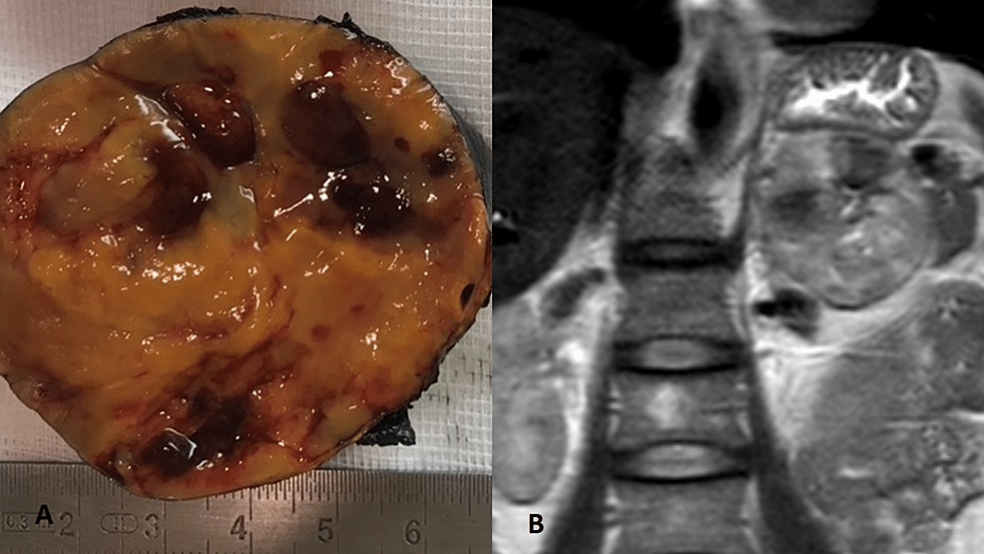
Radiological and pathological correlation. Correlation between gross specimen of an adrenal oncocytoma (A) and the lesion on coronal T2-weighted image of an abdominal MRI.

In conclusion, the study was compatible with an oncocytic neoplasm of the adrenal cortex, and with a Lin-Weiss-Bisceglia Criteria Score of zero, the diagnosis of oncocytoma was favored. The radio-pathology correlation is shown in Figure 7.

## Discussion

Oncocytic neoplasms, or oncocytomas, are usually benign tumors [1], and oncocytic neoplasms of the adrenal gland are extremely rare [2]. Due to scarce literature and consensus, the diagnostic characterization of an adrenal oncocytoma should include both CT and MRI evaluations after the administration of intravenous contrast agents [2].

On CT, usually adrenal oncocytic neoplasms present as round or ovoid, solid, well-defined, and usually encapsulated masses with dimensions around 8 cm [3]. After contrast administration, they tend to have a progressive and heterogeneous enhancement, and they usually lack a central scar, which is seen on most renal oncocytomas [5].

On MRI, adrenal oncocytic lesions appear heterogeneous in both T1 and T2-weighted sequences, with isointense to hypointense signals in T1 and hypersignals in T2 [2,6,7]. The features of DWI sequences vary in studies, usually showing moderate to intense restricted diffusion [5]. In dynamic studies, adrenal oncocytic neoplasm tends to have moderate and progressive contrast uptake [5]. CT and MRI features of adrenal oncocytic neoplasms are nonspecific, making the distinction between oncocytic neoplasms and other benign adrenal lesions a diagnostic challenge.

On pathology, the gross specimen of an adrenal oncocytic neoplasm is a large (around 8 cm) rounded-shape mass, usually encapsulated and well-circumscribed [4]. The matrix of the lesion is brown, yellow, or mahogany, some with hemorrhagic or necrotic areas [4]. The microscopic appearance includes marked eosinophilic cells, rarely with pleomorphic nuclei or mitotic figures [4]. The immunophenotypic profile of the lesions is difficult to evaluate due to a lack of information; usually, an oncocytic tumor is positive for vimentin, melan-A, synaptophysin, and alpha-inhibin [8]. Calretinin is used to differentiate cortical lesions from those of the adrenal medulla, like pheochromocytomas [4]. The Lin-Weiss-Bisceglia Criteria is a morphologic classification proposed to differentiate malignant from benign adrenal oncocytic lesions [3].

The imaging diagnosis of oncocytic adrenal neoplasms can be very challenging, with common overlapping features between other benign lesions or malignant neoplasms, such as lipid-poor adenomas, pheochromocytoma, adrenal metastasis, or adrenal cortical carcinoma.

The imaging feature most commonly used to differentiate benign from malignant adrenal neoplasms is tumor size, because adrenal adenomas usually measure less than 5 cm [4]. However, adrenal oncocytic lesions tend to appear as a large mass, making size criteria not reliable for oncocytic lesions [9].

On imaging modalities, oncocytic neoplasms should be differentiated from adrenal adenomas; heterogeneous appearance, increased attenuation on CT, and, particularly, absence of signal loss on out-of-phase T1-weighted MRI should exclude adenomas, even when lipid-poor [5].

Pheochromocytomas and adrenal oncocytic neoplasm can overlap; however, on imaging modalities, pheochromocytomas tend to be more avidly enhancing, and the results of immunohistochemical staining corroborate the diagnosis [10]. Clinical evaluation can also be very important since most pheochromocytomas are functional, as opposed to oncocytic lesions [10].

The distinction between benign adrenal oncocytic neoplasms and adrenocortical carcinomas on imaging modalities is very difficult; however, adrenocortical carcinomas at presentation usually have distant metastasis (mostly liver and lung), adjacent tissue invasion, and adenopathies, especially in large tumors [2].

Large adrenal lesions are an indication for surgical resection, usually laparoscopic adrenalectomy, if preoperative imaging reveals an encapsulated tumor with no signs of invasion of adjacent tissues or regional adenopathies [2,4].

Accurate pathological classification of adrenal oncocytic neoplasms is crucial for the patient's prognosis, and it is based upon the Lin-Weiss-Bisceglia Criteria [3]. Most adrenal oncocytic lesions are benign and can be assessed conservatively with a radiological follow-up of five years, usually with no signs of recurrence [4].

## Conclusions

Adrenal oncocytomas are an extremely uncommon diagnosis. Radiological and pathological diagnosis is challenging due to the overlap of features with other benign or malignant lesions. Oncocytic neoplasms should be considered in the differential diagnosis of a large, indeterminate adrenal tumor. An adrenal oncocytoma is usually an indication for surgical resection, when large in size, usually with no recurrence on imaging follow-up of five years.
